# To Angulate or Not to Angulate the Ulna during the Progressive Distraction Period Performed with a Monolateral External Fixator in Paediatric Patients with a Chronic Monteggia Fracture?

**DOI:** 10.3390/medicina58111666

**Published:** 2022-11-17

**Authors:** Yanhan Liu, Hai Zhao, Hongwen Xu, Weizhe Shi, Jingchun Li, Yiqiang Li, Federico Canavese

**Affiliations:** 1 Guangzhou Women and Children’s Medical Center, Guangzhou 510623, China; 2 Chenzhou No.1 People’s Hospital, Chenzhou 423000, China; 3 Faculty of Medicine, Lille University Hospital, 59000 Lille, France

**Keywords:** chronic Monteggia fracture, ulna osteotomy, distraction, angulation, children

## Abstract

Background and Objectives: The purpose of this study was to compare the clinical and radiographic evolution of chronic Monteggia fractures (CMFs) treated by ulnar osteotomy and monolateral external fixators (MEFs) with or without angulation of the ulna during the distraction period. Materials and Methods: This retrospective study evaluated 20 children (14 boys and 6 girls) with CMFs. According to the strategy of ulnar lengthening, two groups of patients were identified: patients undergoing gradual lengthening with (Group A, *n* = 11) or without ulna angulation (Group B, *n* = 9). The mean age at the time of surgery was 7.7 years old (range, 5.4–12.9). The mean time from initial trauma to surgery was 26.3 months (range, 1–96), and the mean follow-up was 24.6 months (range, 5.5–45.4). Clinical outcomes were evaluated by Kim et al.’s Elbow Performance Score, while radiographic outcomes were assessed on plain radiographs. Results: Age at surgery, sex, laterality, time between trauma and surgery, and time of follow up in the two groups of patients showed no significant differences. The radial head was successfully reduced in 9 of 9 and 10 of 11 patients in Groups B and A, respectively (*p* = 1.00). The mean time to achieve radial head reduction was shorter in Group B (18.1 ± 5.3 days) than in Group A (39.2 ± 18.7 days; *p* = 0.004). The mean angulation of the ulna at the end of treatment was significantly lower in Group B (0.6° ± 1.1°) than in Group A (25.9° ± 6.3°; *p* < 0.0001). The average ulnar lengthening at the end of treatment in Group B (14.1 ± 5.8 mm) was, on average, 7.7 mm less than that in Group A (21.8 ± 9.7 mm; *p* = 0.05). The Kim et al. Elbow Performance Score at the last follow-up visit was comparable between the two groups of patients (*p* = 1.00). Conclusions: A shorter time to achieve radial head reduction and less deformity of the ulna can be expected in paediatric patients with CMFs undergoing intraoperative restoration of ulnar alignment and gradual lengthening without angulation postoperatively.

## 1. Introduction

Monteggia fracture, described by Giovanni Battista Monteggia in 1814, is characterized by a fracture of the ulna associated with dislocation of the proximal radius [1]. In cases in which the initial radiographs do not include the elbow joint, or there is a subtle greenstick or plastic deformity of the ulna, the fracture can be easily missed [2,3,4]. Failure to diagnose an acute Monteggia injury, and when proximal radius dislocation persists for more than 4 weeks, delineates a chronic Monteggia fracture (CMF) [5]. In the presence of a CMF, some authors have recommended an ulnar osteotomy and open reduction of the radial head, associated or not with reconstruction of the annular ligament [6,7,8,9,10], while others have suggested closed and “indirect” reduction of the radial head by osteotomy of the ulna and its angulation, progressive or immediate [11,12,13].

The treatment of CMFs is challenging and is characterized by relatively unpredictable outcomes. In addition, the best treatment method for this type of injury has yet to be identified and remains a source of debate among specialists [2,14,15,16].

Since 2015, at two institutions in China, we have been managing patients with CMFs according to the technique described by Exner in 2001, which allows for closed reduction of the radial head by elongation and angulation of the ulna using a monolateral external fixator (MEF) [17]. A variant of the method described by Exner has been used since 2018 at our institution. As of this date, patients with CMFs were treated with intraoperative restoration of ulnar alignment and its gradual lengthening without making any angulation, on either the frontal or the sagittal plane.

The purpose of this study was to compare the clinical and radiographic evolution of CMFs treated by ulnar osteotomy and MEF, with or without angulation of the ulna during the distraction period. The hypothesis was that the two lengthening procedures, with or without ulna angulation, would allow for reduction of the dislocated radial head in children with CMFs.

## 2. Materials and Methods

Following Institutional Review Board approval, we retrospectively reviewed all children with CMFs treated at two institutions between January 2015 and December 2020 according to the reported techniques.

The inclusion criteria were as follows: (1) confirmed diagnosis of CMF (time between trauma and surgery >4 weeks) [5]; (2) surgical treatment by ulna osteotomy, MEF and distraction with or without angulation of the ulna; (3) follow-up >6 months; and (4) complete radiological and clinical data. Patients with an ipsilateral upper extremity soft tissue tumour or congenital radial head dislocation were excluded.

Twenty patients with a CMF who were treated by ulnar osteotomy and gradual reduction of the proximal radius using an MEF were identified and divided into two groups. Eleven patients underwent gradual distraction and angulation of the ulna (Group A), while nine underwent restoration of ulnar alignment intraoperatively and gradual lengthening along the axis of the ulna postoperatively (Group B). Table 1 summarizes the patient demographics and surgical procedures (Table 1).

### 2.1. Surgical Technique

One linear 1.5- to 2-cm skin incision was made distally to the tip of the olecranon, the proximal ulna was exposed, and a transverse osteotomy was performed. Two types of external fixators were used according to the different modalities of ulna distraction (Orthofix, Model M511 for Group A, and Model 55010 for Group B). Bicortical pins were introduced under fluoroscopy proximally and distally to the osteotomy site and perpendicularly to the main axis of the ulna; the pins were then linked to the MEF.

The type of treatment, distraction with or without angulation of the ulna, was based on the surgeon’s preference.

**Figure 1:**
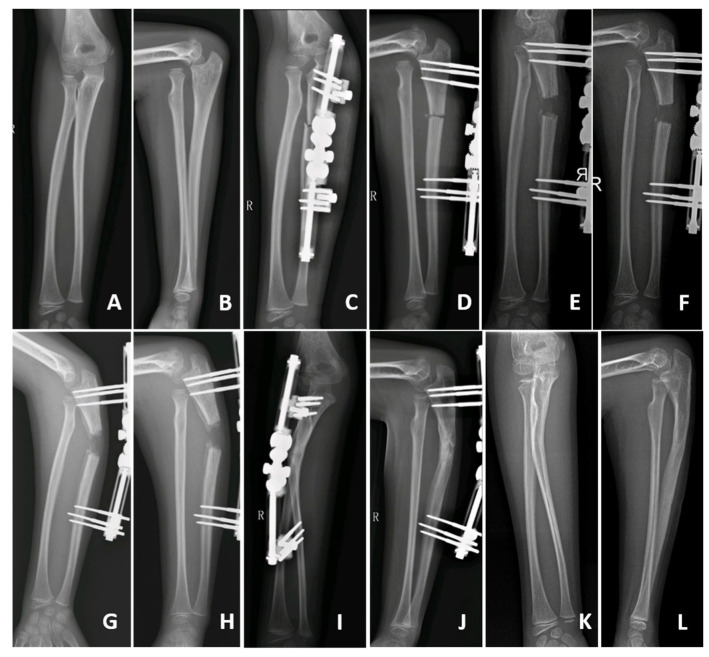
A 7-year-old girl managed by gradual lengthening and angulation of the ulna (patient no. 3, Group A): preoperative (**A**,**B**), postoperative (**C**,**D**), gradual lengthening (**E**,**F**), gradual angulation (**G**,**H**), healing of the ulna (**I**,**J**), final follow-up (**K**,**L**).

In Group A patients, 7 days after osteotomy and MEF application, the proximal ulna was gradually distracted (1 mm per day) and dorsally angulated until satisfactory reduction of the radial head was obtained. In particular, the angulation of the ulna was performed without preestablished limits and continued until a satisfactory reduction was achieved (Figure 1).

**Figure 2:**
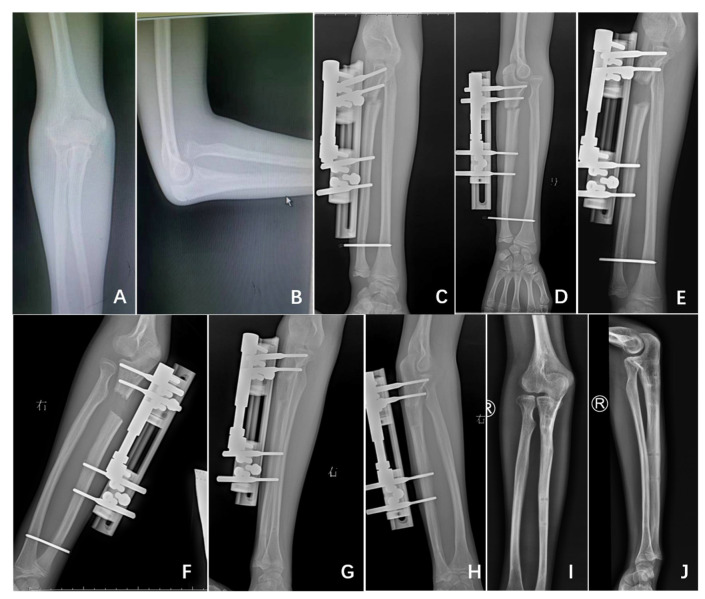
A 13-year-old boy treated by gradual lengthening of the ulna without angulation (patient no. 12, Group B); preoperative (**A**,**B**), postoperative (**C**,**D**), gradual lengthening (**E**,**F**), healing of the ulna (**G**,**H**), final follow-up (**I**,**J**).

In Group B patients, in contrast, at the time of the index procedure, ulnar alignment was restored and verified by fluoroscopy intraoperatively [rectilinear on anterior-posterior (AP) and lateral images], and the distal radioulnar joint was stabilized by a 2-mm K-wire. After a period of 7 days, gradual distraction of the ulna, without any angulation, was performed at a rate of 1 mm per day until the reduction of the proximal radius was achieved (Figure 2).

### 2.2. Radiological Evaluation

Full-length AP and lateral radiographs of the injured forearm, including the elbow and wrist, were obtained: (i) at the time of injury to evaluate the location of the fracture and the direction of the dislocation according to Bado’s classification [20]; (ii) every 7 days during the distraction phase to assess the evolution of the reduction; and (iii) every 3 to 6 months thereafter to evaluate bone healing and to monitor the stability of the reduction.

**Figure 3:**
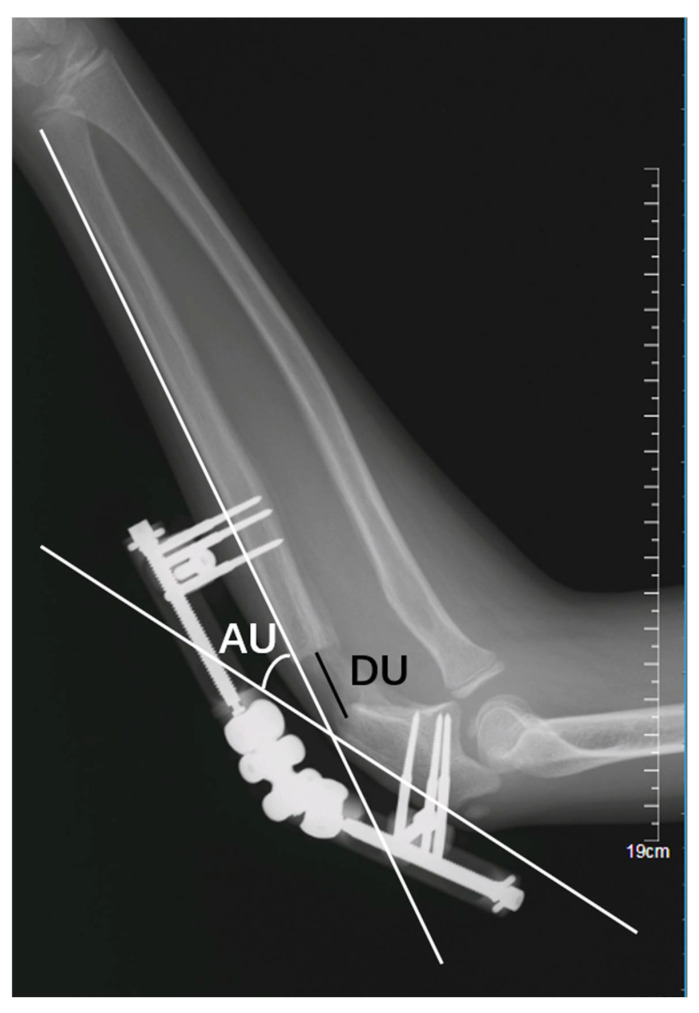
Lengthening distance (DU) and angulation of the ulna (AU).

The following parameters were then measured on lateral radiographs at the end of treatment:

(1) Lengthening distance of the ulna (DU): the distance, in millimetres (mm), between the proximal and distal fragments of the fractured ulna (Figure 3); and
(2) Angulation of the ulna (AU): the angle, in degrees, between the line passing at the level of posterior cortex of the proximal and the distal fragment of the fractured ulna (Figure 3);

### 2.3. Clinical Outcomes

All patients underwent regular follow-up for 24.6 months on average (range, 5.5–45.4). At the last follow-up visit, clinical outcome was assessed using the Kim et al. Elbow Performance Score [18,19] (Table 2).

### 2.4. Statistical Analysis

The continuous data of the two groups were described by means ± standard deviations (SDs), and their differences were compared with the independent-samples *t* test. Categorical data are presented as percentages and were compared with the χ^2^ test. All tests were two sided with a significance level of 0.05 and were performed using SPSS software (version 22.0; Armonk, NY: IBM, USA).

## 3. Results

A total of 20 children (14 boys, 6 girls) with CMFs managed by the reported techniques were reviewed. The right side was involved in 14 cases (70%), and the left side was involved in 6 cases (30%). There were 19 Bado type I and 1 Bado type II fractures (5%; patient n.15, Group B) [20]. The mean age at the time of injury was 6.2 years old (range, 2 to 12), and the mean interval between injury and index surgical procedure was 24.2 months (range, 1 to 96). The mean follow-up time was 24.6 months (range, 5.5–45.4). No differences existed between the two groups with respect to age at surgery, sex, laterality, time between trauma and surgery, or length of follow-up (Table 3).

### 3.1. Radiological Evaluation

Eleven of twenty patients (Group A; 55%) underwent gradual angulation and lengthening of the ulna with MEF. The mean duration of correction was 39.2 days (range, 14–75); the mean angulation and lengthening of the ulna at the end of treatment were 25.9° (range, 17–36°) and 21.8 mm (range, 8–42), respectively.

Nine of twenty patients (Group B; 45%) underwent restoration of ulna alignment intraoperatively and gradual lengthening postoperatively at a rate of 1 mm/day with MEF. The mean duration of treatment was 18.1 days (range, 12–28); the mean angulation and lengthening of the ulna at the end of treatment were 0.6° (range, 0–3°) and 14.1 mm (range, 8–25), respectively.

The average length of time to achieve radial head reduction was 21.1 days faster in Group B than in Group A (18.1 ± 5.3 days versus 39.2 ± 18.7 days; *p* = 0.004); the average ulnar angulation at the end of treatment was on average 13.2° lower in Group B than in Group A (0.6° ± 1.1° versus 25.9° ± 6.9°; *p* < 0.001). The average ulnar lengthening at the end of treatment in Group B was, on average, 7.7 mm less than that in Group A (14.1 ± 5.8 mm versus 21.8 ± 9.7 mm; *p* = 0.05) (Table 3).

The radial head was successfully reduced in all patients in Group B (9/9.100%), the reduction was maintained at the time of MEF removal, and no recurrence was observed at the last follow-up visit. In Group A, however, the radial head was successfully reduced in 10 of 11 (90.9%) patients, and no recurrence of dislocation was observed at the last follow-up visit (*p* = 1.00).

### 3.2. Clinical Evaluation

The Kim et al. Elbow Performance Score [18,19] at the last follow-up visit was excellent in all Group B cases. The results were similar in Group A patients, in whom the score was excellent in 10 children, fair in 0, and poor in 1; *p* = 1.00). The patient rated as poor complained of intermittent elbow pain and reduced elbow flexion (−30°) due to insufficient radial head reduction (patient no. 10, Group A).

## 4. Discussion

Our study found that intraoperative restoration of ulnar alignment and progressive lengthening of the ulna without angulation during the postoperative period constitute an effective method for the treatment of CMFs; moreover, this technique requires less time to achieve reduction of the radial head and results in an ulna with much less deformity than is found in patients managed with lengthening and angulation.

As a challenging condition that is difficult to treat, several procedures to achieve and maintain radial head reduction have been described to manage patients [6,9,14,21,22]. Compared to open reduction, gradual closed reduction of the radial head is less invasive. Kim et al. reported that soft tissue contracture and a tight interosseous membrane and biceps tendon contribute to proximal migration of the radius and prevent reduction of the radial head [18]. In this respect, gradual modification of the ulna allows for progressive reduction of the radial head and avoids tensioning of the soft tissue structures, including the interosseous membrane and the biceps tendon; moreover, the technique does not require open reduction of the radial head or annular ligament reconstruction.

Eleven of twenty patients (Group A) were treated by gradual distraction and dorsal angulation of the ulna, as described by Exner and Bor et al. [17,23]. In Group A patients, the mean ulna angulation was 25.9°, which is comparable to the data reported by Bor et al. (26° ± 6.3°) [23] and Yuan et al. (22.9° ± 7.7°) [12]. Therefore, gradual lengthening and angulation of the ulna can compensate for the length discrepancy and can maintain the reduction of the radial head through the stabilizing action of the interosseous membrane, which appears to be important to maintaining the radial head in the correct position [24]. However, excessive dorsal angulation of the ulna can create some drawbacks, such as deformity, unaesthetic appearance of the proximal forearm and, most importantly, limitations in prone supination. Although ulnar deformities can remodel during childhood growth, it can induce apprehension in the patient and his or her family; in older children, moreover, complete remodelling might not occur.

In Group B patients (*n* = 9), a technique based on progressive ulnar lengthening [17,23] but without any angulation was used. In these patients, we performed osteotomy at the level of the apex of the ulnar deformity and restored its alignment at the same time. A K-wire was then used to stabilize the distal end of the radius and ulna. Then, after one week, gradual lengthening of the ulna was performed, preserving the tension of the interosseous membrane, which might contribute to keeping the radial head reduced. Previous research has demonstrated that, in patients with CMFs, the length of the ulna on the injured side is significantly shorter than that on the normal side [25]. Huang et al. reported that the average ratio between ulnar and radial length in normal children is approximately 1.1 [26]. We hypothesized that stable radial head reduction could only be achieved if the ulna/radius ratio was restored after correction of ulnar alignment [25,26]. In particular, the radial head could be successfully reduced in all patients (9/9, 100%), and no recurrence was observed during follow-up; in addition, the time to achieve reduction of the radial head was significantly shorter. Among Group A patients, conversely, although no recurrences were observed, reduction was not possible in one case.

Although several surgical treatment modalities have been described, ulnar osteotomy remains essential in the management of CMFs. A review of 30 studies that included a total of 600 patients showed that performing a proximal ulnar osteotomy is the most significant predictor of eventual radial head reduction [27]; however, the choice of osteotomy site remains controversial. At the level of the apex of the ulnar deformity and at the proximal part of the ulnar metaphysis are the most frequently cited osteotomy sites, and the problem of nonunion or delayed union is most evident at the level of apical osteotomies [28,29]. In Group B patients, we performed the osteotomy at the level of the apex of the ulnar deformity. From our data, no cases of nonunion or delayed union during the lengthening procedure were identified. However, all types of lengthening require regular radiographs to monitor the healing of the osteotomy and to avoid overstretching of the ulna, which could lead to nonunion/delayed union [30,31,32].

We encountered some limitations in the analysis of our results. First, there were intrinsic limitations related to the retrospective nature of our study. Second, the sample size was relatively small; however, CMFs are rare, and this study is the first attempting to compare two different lengthening modalities of the ulna. Third, it is possible that further complications could develop in the future; however, the mean follow-up was over 2 years, and all of the patients underwent functional and radiographic assessment by validated tools.

## 5. Conclusions

In conclusion, a shorter time to achieve radial head reduction and fewer deformities of the ulna can be expected in CMF patients undergoing intraoperative restoration of ulnar alignment and gradual lengthening without angulation postoperatively. Ulna angulation might not be systematically needed in children with CMFs treated by MEF, and gradual distraction could restore ulnar alignment.

## Figures and Tables

**Table 1:** Treatment modality and outcome (*n* = 20).

Patient	Treatment	Duration of Distraction (Days)	External Fixator Modifications	AU (Degrees)	LU (mm)	Radial Head Reduction	Redislocation	Kim et al. Elbow Performance Score [18,19]
1	UO+GLA	41	Days 7–26: L Days 27–48: A	36	19	Yes	No	Excellent
2	UO+GLA	14	Days 7–21: L+A	21	16	Yes	No	Excellent
3	UO+GLA	28	Days 7–22: L Days 23–29: A Days 30–35: L	18	19	Yes	No	Excellent
4	UO+GLA	18	Days 7–10: L+A Days 11–25: L	23	9	Yes	No	Excellent
5	UO+GLA	21	Days 7–21: L Days 22–28: A	25	12	Yes	No	Excellent
6	UO+GLA	48	Days 7–41: L Days 42–55: A	17	42	Yes	No	Excellent
7	UO+GLA	49	Days 7–27: L Days 28–42: A Days 43–56: L	26	22	Yes	No	Excellent
8	UO+GLA	75	Days 7–14: L Days 15–21: L+A Days 22–82	27	36	Yes	No	Excellent
9	UO+GLA	52	Day 0: A Days 7–59: L+A	35	23	Yes	No	Excellent
10	UO+GLA	30	Days 7–13: A Days 14–37: L	25	17	Dislocated	-	Poor
11	UO+GLA	55	Day 0: A Days 7–62: L	32	25	Yes	No	Excellent
12	UO+GL	20	Days 7–27: L	0	18	Yes	No	Excellent
13	UO+GL	18	Days 7–25: L	0	12	Yes	No	Excellent
14	UO+GL	18	Days 7–25: L	0	11	Yes	No	Excellent
15	UO+GL	22	Days 7–29: L	0	25	Yes	No	Excellent
16	UO+GL	10	Days 7–17: L	0	10	Yes	No	Excellent
17	UO+GL	28	Days 7–35: L	3	21	Yes	No	Excellent
18	UO+GL	18	Days 7–25: L	0	8	Yes	No	Excellent
19	UO+GL	12	Days 7–19: L	0	11	Yes	No	Excellent
20	UO+GL	17	Days 7–24: L	2	11	Yes	No	Excellent

UO, ulnar osteotomy; GLA, gradual lengthening and angulation of the ulna; GL, gradual lengthening of the ulna; AU, angulation of the ulna; LU, lengthening of the ulna.

**Table 2:** Clinical outcome according to Kim et al. Elbow Performance Score [18,19].

Parameters	25 Points	15 Points	0 Points
Deformity	No concern	Minor concern	Major concern
Pain	No pain	Intermittent mild pain but no limit to activities	Pain
Range of movement *	>250°	200° to 250°	<200°
Function	Five activities of daily living: combing hair, feeding self, opening doorknob, grabbing the high object, putting on shoes with hands	Five points for each activity	
No. of patients/outcome	15/good	2/fair	2/poor

* Range of movement is the sum of the flexion-extension and pronation-supination degree; full flexion–extension was defined as 140°, full pronation as 75°, and full supination as 85°.

**Table 3:** Patients managed by gradual lengthening with (Group A) or without ulna angulation (Group B).

	Group A (*n* = 11)	Group B (*n* = 9)	*p* Value
Gender (male/female)	8/3	6/3	1.00
Laterality (left/right)	3/8	3/6	1.00
Age at surgery (year)	8.1	7.2	0.40
Time between trauma and surgery (months)	23.3	30	0.60
Follow-up (months)	20.8	29.4	0.10
The duration of radial head reduction	39.2	18.1	0.004
Mean angulation of the ulna (degrees)	25.9	0.6	0.00
Mean lengthening of the ulna (mm)	21.8	14.1	0.05
Kim et al. Elbow Performance Score			1.00
Excellent	10	9	
Good	0	0	
Fair	0	0	
Poor	1	0	

## Data Availability

All the available data have been presented in this study. Details regarding the data supporting the reported results can be requested at the following e-mail address: xuhongwen@gwcmc.org.
